# Metagenomic next-generation sequencing in the diagnosis of leptospirosis presenting as severe diffuse alveolar hemorrhage: a case report and literature review

**DOI:** 10.1186/s12879-021-06923-w

**Published:** 2021-12-07

**Authors:** Meiqin Chen, Weili Lu, Shugen Wu, Shun Wang, Tao Lu, Chunxian Peng

**Affiliations:** Department of Infectious Diseases, The Quzhou Affiliated Hospital of Wenzhou Medical University, Quzhou People’s Hospital, Quzhou, 324000 Zhejiang China

**Keywords:** Severe diffuse alveolar hemorrhage, Leptospirosis, Metagenomic next-generation sequencing, Case report

## Abstract

**Background:**

Leptospirosis is a common infectious disease in tropical and semitropical regions, and it is typically neglected. Leptospirosis-associated acute diffuse alveolar hemorrhage is one of its fatal complications. The use of bronchoalveolar lavage fluid (BALF) metagenomic next-generation sequencing in the diagnosis of *Leptospira interrogans* infection has rarely been reported.

**Case presentation:**

We present the case of a 62-year-old female who was transferred to our hospital with dyspnea, and severe hemoptysis and was supported by a tracheal intubation ventilator. Bronchoalveolar lavage fluid (BALF) metagenomic next-generation sequencing (mNGS) reported *Leptospira interrogans*. A diagnosis of diffuse alveolar hemorrhage caused by leptospirosis was made. After immediately receiving antibiotics and hormone therapy, the patient achieved a complete recovery upon discharge.

**Conclusion:**

Leptospirosis presenting as severe diffuse alveolar hemorrhage is rare but should be considered in the differential diagnosis. mNGS can help identify pathogens and treat them early, which can improve prognosis.

## Background

Leptospirosis is usually acknowledged to be the most common animal disease in the world caused by Leptospira [1]. Humans become infected once mucous membranes or broken skin come into contact with water, soil or direct contact with bodily fluids of infected animals [2]. Leptospirosis has different clinical manifestations without obvious specificity, and it is difficult to identify. In China, the earliest leptospirosis case could be traced to the 1930s. *Leptospira interrogans* is considered to be a leading cause of leptospirosis, although other pathogenic species have also been found [3]. With numerous rivers and lakes, a moist climate, and rice-planting tradition, these areas confer advantages for the spread and prevalence of *Leptospira interrogans *[4]. The gold standard for leptospirosis diagnosis is a MAT to detect antibodies to Leptospira. It is difficult to achieve a timely and accurate diagnosis. In recent years, mNGS has been applied to clinical samples and improves the diagnostic yield of rare pathogens. As early as 2014, it was reported that the next-generation gene sequencing could diagnose Leptospira neuropathy [5]. In this case, we describe a patient with severe diffuse alveolar hemorrhage caused by leptospirosis.

## Case presentation

**Fig. 1:**
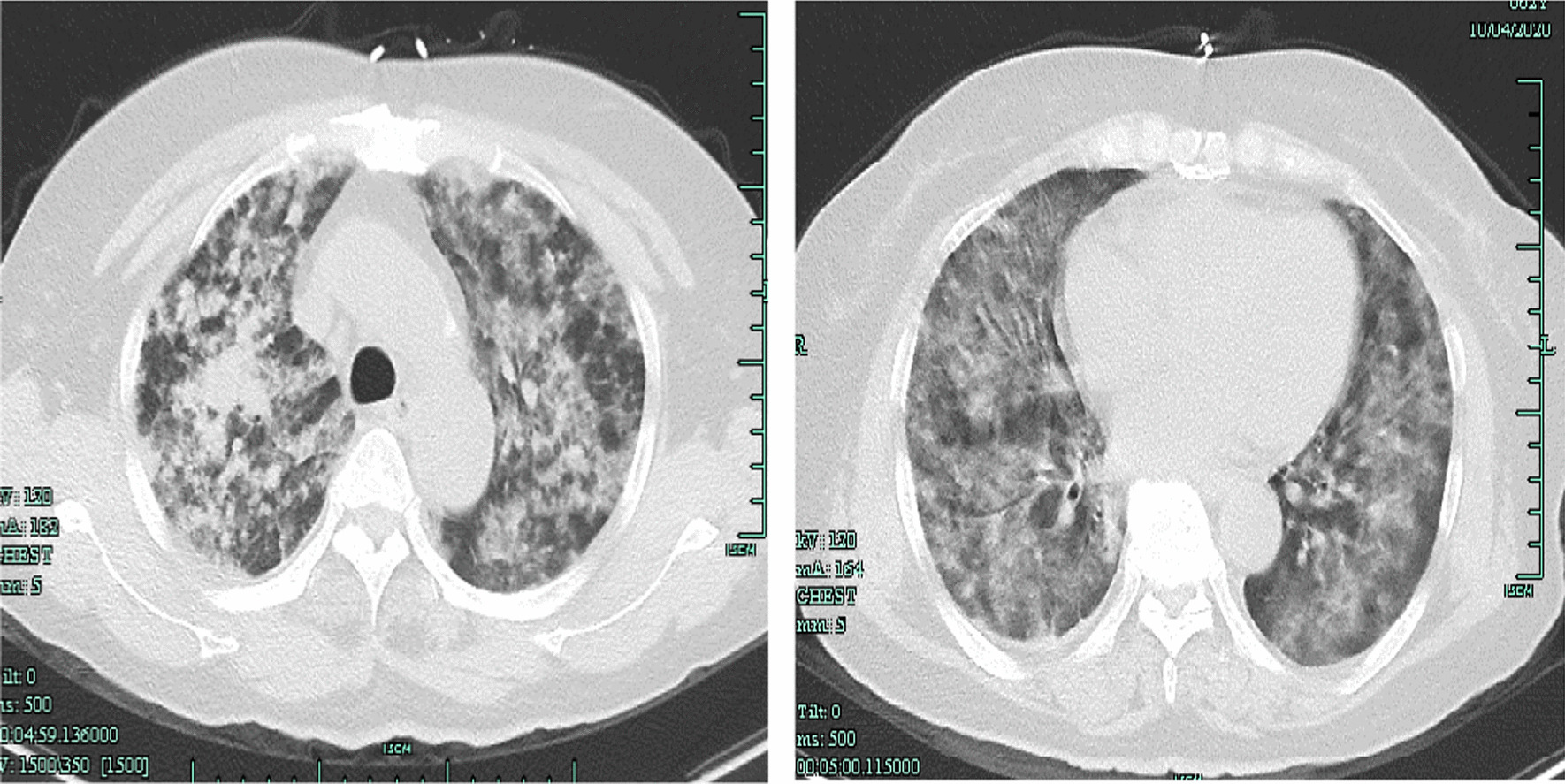
Chest CT showing diffuse alveolar infiltrates with acute respiratory failure on admission

A 62-year-old female presented with a 5-day history of myalgia, fatigue, and vomiting. Two days before she came to the hospital, she developed dyspnea, and nine hours before she came to the hospital, she developed hemoptysis. She denied travel to any areas of endemic. She had 20-year hypertension history. Physical examination revealed high blood pressure (160/70 mmHg), a pulse rate of 113 beats per minute and oxygen saturation of 94% on oxygen mask air, and tachypnea with signs of respiratory distress, no conjunctival suffusion and no icteric sclera. Soon after admission, the patient had blood gas analysis suggesting an oxygenation index of 114, necessitating endotracheal intubation and ventilator support. She required pressure control ventilation (PCV) to maintain adequate oxygenation with high PEEP. She remained tachycardic but was otherwise hemodynamically stable. Her chest CT showed bilateral alveolar infiltrates (Fig. 1). Clinical investigations demonstrated anemia, hypoproteinemia and thrombocytopenia, with normal renal function and liver function, without jaundice (Table 1). With the progression of the clinical course, pulmonary hemorrhage was unusual and led to a diagnostic dilemma in view of the etiology. The diagnosis was considered vasculitis or tuberculosis. Tests for anti-nuclear antibodies and anti-neutrophil cytoplasmic antibodies were negative, and complement levels were normal. Sputum was negative for acid-fast bacilli, and a GeneXpert assay was negative. After mechanical ventilation, the patient's hemoptysis did not improve significantly, there was severe hypoxemia, the ventilator settings were high, and the oxygenation index was poor. Therefore, we performed bronchoscopy, and at the same time, we collected BALF and performed next-generation gene sequencing. While waiting for the results, we give the patient a comprehensive treatment with adequate sedation, analgesia, and hemostasis as well as piperacillin-tazobactam antiinfection therapy. Two days later, the mNGS results showed *L. interrogans* infection (Table 2). Negative hepatitis serology, dengue NS1 antigen and antibodies, and serology for *Mycoplasma pneumoniae* excluded other possible infective pathologies. One week later, the CDC (Centers for Disease Control and Prevention) of Zhejiang Province responded with positive detection of anti-Leptospira IgG in a microscopic agglutination test (MAT) for leptospirosis, elucidating the clinical picture.

**Table 1:** Laboratory results

Test	4/10/20	5/10/20	6/10/20	12/10/20	22/10/20
Potassium (mmol/l)	3.94	3.2	3.78	3.59	3.48
Urea (mmol/l)	11.02	13.94	11.42	7.09	2.72
Creatinine (mmol/l)	92.6	61.6	58.8	60.5	42.8
Bilirubin (µmol/l)	8.4	12.6	17.5	18.5	8.6
Alk phos (U/l)	69.7	75.1	77.1	68.5	85.6
ALT (U/l)	22.7	16.5	17.4	17.8	13.5
Total protein (g/l)	40.7	49.5	54.1	46.5	52.5
Albumin (g/l)	20.9	27.5	31.7	27.8	29.9
CRP (mg/l)	132.88	116.48	80.65	2.12	4.38
HB (g/dl)	66	74	83	86	88
WCC (× 10^9^/l)	13.3	5.0	8.7	12.6	4.4
PLT (× 10^9^/l)	81	96	97	136	159
Neut (× 10^9^/l)	12.36	7.85	8.44	11.24	2.68
Lymph (× 10^9^/l)	0.18	0.35	0	0.87	0.73
FiO_2_ Air	100% + PCV	80% + PCV	80% + PCV	40%	30%
PEEP	10	8	6		
pH	7.397	7.352	7.366	7.463	7.475
pO_2_	77.9	61.8	79.1	101	105
pCO_2_	30.4	35.9	38.9	36	35.5
Lactic acid	0.6	1.2	1.5	0.71.1

**Table 2:** Pathogenic microorganisms detected by using next-generation sequencing

Genus	Species	Number of detected reads
Leptospira	*Leptospira interrogans*	4

**Fig. 2:**
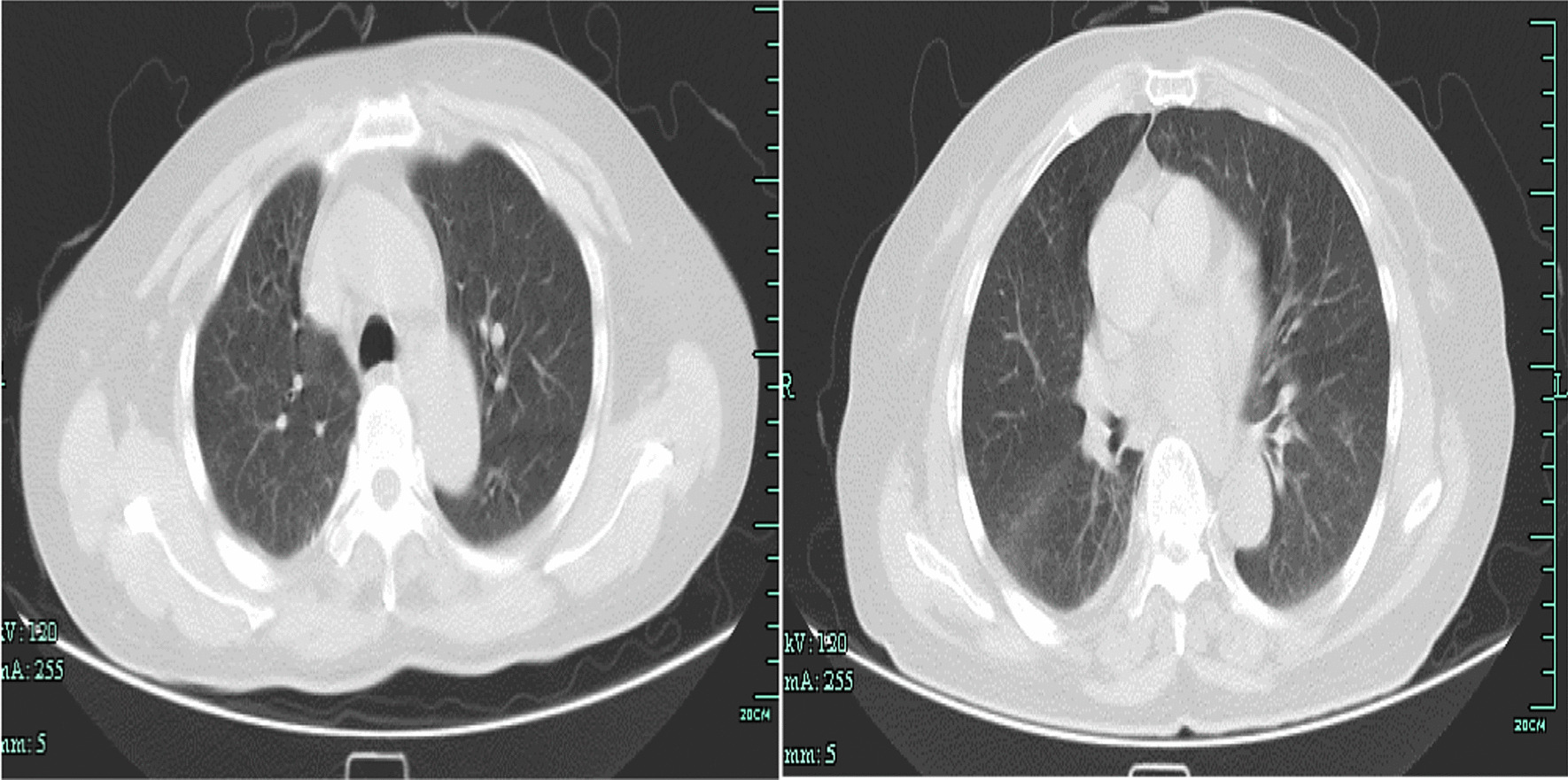
Chest CT showing exudation and absorption before discharge

Intravenous penicillin G every 6 h was currently applied and continued for 2 weeks together with supportive care. Ventilator support was continued for 8 days. The patient improved dramatically, and reexamination of chest CT exudation lesions showed obvious absorption (Fig. 2). Once the diagnosis was established and explained to the patient, she become aware of how she had developed the infection: the week before becoming ill, she had worked in the rice fields.

### Metagenomic NGS

**Fig. 3:**
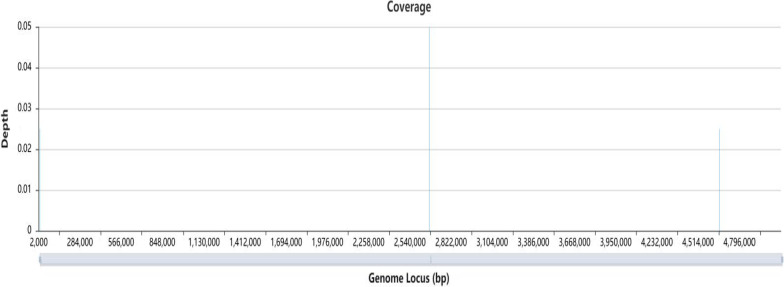
The coverage of the Leptospira reference genome of was 3.9e−3%

High-quality sequencing data were obtained by microbial cell wall disruption, fully automatic nucleic acid extraction, and PCR-free (no amplification) library construction technology, and the reads were aligned with Microbial Genome Databases, which contained 6375 whole-genome sequences of viral taxa, 9204 bacterial genomes or scaffolds, 472 fungal genomes, and 149 parasite genomes related to human infectivity. After receiving the results of taxonomic assignments, we aligned reads mapped to *L. interrogans* by MegaBLAST to the NT database with default parameters for further confirmation (Fig. 3).

## Discussion and conclusions

Leptospirosis typically presents with clinical features of flu-like symptoms with fever, myalgia, conjunctivitis and mild gastrointestinal discomfort, followed by multiorgan damage that may be complicated by jaundice, renal failure, pulmonary hemorrhage, acute respiratory distress syndrome, and other complications [6]. Leptospirosis with pulmonary involvement may present chest pain, cough, dyspnea, hemoptysis, ARDS, and diffuse bilateral bronchoalveolar infiltrates involving all lobes, with mortality rates reported to be as high as 75% [7, 8]. Patients with pulmonary hemorrhage often suffer from hypoxemia, which is resistant to mechanical ventilation and has proven particularly difficult to treat. The pathogenesis of pulmonary hemorrhage is not yet fully understood. It is thought to be associated with cytotoxic factors in the tissue, especially in the liver and kidney, and host immune mechanisms, particularly in the lungs [1, 9]. As previously noted [10], hemorrhage is due to primary noninflammatory vasculopathy. This mechanism may be related to a reduction in CD34 levels and retention of aquaporin 1 expression. A recent study revealed [11] that vWA and platelet-activating factor acetylhydrolase-like protein from *L. interrogans* will cause severe pulmonary hemorrhage in mice.

Leptospirosis is sometimes a self-limiting disease, however early use of antibiotics will shorten the duration of disease, reduce severity and expedite recovery. Treatment should be started before serologic affirmation. In this case, pulmonary hemorrhage was significantly reduced after penicillin was used, and the ventilator conditions gradually declined. Finally, the patient was weaned from the ventilator for successful extubation. MAT is considered the ‘gold standard’ test for diagnosis; however, it does not permit early diagnosis because it relies on the detection of antibodies to leptospiral antigens and cannot detect infection until 5–7 days after exposure. Recent developments in mNGS have helped elucidate organisms and infections at the molecular level [12, 13]. mNGS is appropriate for the detection of pathogens that cannot be identified by other existing detection techniques for rare and slow-growing bacteria, for which it is difficult to obtain pathogenic bacteria from conventional culture [5, 14, 15]. mNGS offers considerable advantages in shortening the time needed for diagnostic confirmation of bacterial/fungal infection, promoting targeted antimicrobial treatment, and improving patient prognosis [16]. BALF collection is well tolerated and safely performed in acutely ill patients [17]. BALF mNGS improves the capacity of pathogen detection and provides guidance in the clinic, which is easy to implement in practice [18, 19]. However, mNGS findings should be combined with epidemiological and clinical characteristics before a pathogenic microbe can be identified. No other bacteria were detected by BALF mNGS, and *L. interrogans* was found. Even though there were only 4 reads, despite low gene coverage, based on the patient's rice field operation history, pulmonary hemorrhage and mNGS, leptospirosis was diagnosed in time, and penicillin was given promptly. After effective early treatment, the patient was transferred from the critically ill to the general infection department for continued treatment and was discharged after 1 week.

In conclusion, leptospirosis with pulmonary hemorrhage has proven to be particularly difficult to treat. Early clinical suspicion and laboratory confirmation of leptospirosis are crucial since delayed diagnosis may increase mortality. mNGS played an important role in this case and was a powerful and rapid tool for diagnosis of atypical manifestations.

## Data Availability

The data that support the findings of the current study are available from the corresponding author upon reasonable request. The *L. interrogans* sequencing data used in this study are available in the Sequence Read Archive under SRA accession number SRR13780073.

## Abbreviations

NGS: Next-generation sequencing
mNGS: Metagenomic next-generation sequencing
BALF: Bronchoalveolar lavage fluid
MAT: Microscopic agglutination test
CT: Computed tomography
